# Supplementary material for: Regulatory mechanisms of incomplete huntingtin mRNA splicing
Source: Nat Commun. 2018 Sep 27;9:3955. doi: 10.1038/s41467-018-06281-3 (PMC6160442; doi:10.1038/s41467-018-06281-3)
Supplement: Supplementary file 1 — Supplementary Information [file 41467_2018_6281_MOESM1_ESM.pdf]

Supplementary Information

Regulatory mechanisms of incomplete huntingtin mRNA splicing

Andreas Neueder[1,2]*, Anaelle A. Dumas[1], Agnesska C. Benjamin[1], Gillian P. Bates[1]*

[1]UCL Huntington's Disease Centre, Department of Neurodegenerative Disease and Dementia Research Institute, UCL Institute of Neurology, University College London, London, United Kingdom

[2]Current address: Department of Neurology, Ulm University, Ulm, Germany

*Correspondence:

Andreas Neueder andreas.neueder@uni-ulm.de

Gillian P. Bates gillian.bates@ucl.ac.uk

SUPPLEMENTARY FIGURES

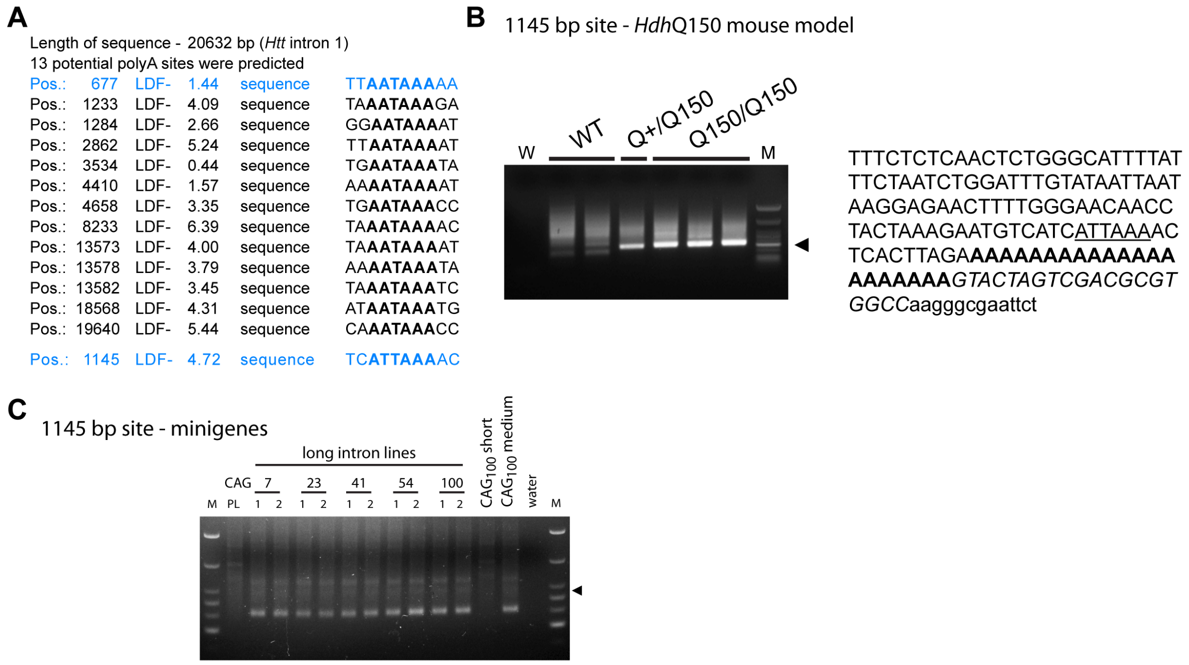

Supplementary Fig. 1. A second cryptic polyA site, 1145 bp into intron 1 of *Htt*, is used in the *Hdh*Q150 mouse model.

(**A**) Cryptic polyA sites (SoftBerry polyAH algorithm) in intron 1 of mouse *Htt*. The site at 1145 bp contains the minor core polyA site sequence ATTAAA. (**B**) Left panel: The 3'RACE product (◄) at 1145 bp into intron 1 of *Htt* (see also Fig. 1H) was amplified from cDNA (primers 1145 and 1145nest, see Supplementary Table 1), which was reverse transcribed from total RNA extracted from cortices of wild type (WT), heterozygous (Q+/Q150) and homozygous (Q150/Q150) *Hdh*Q150 mice at 2 months of age. W = water; M = low molecular weight DNA ladder (New England Biolabs). Right panel: Sequencing of the 3'RACE product confirmed the identity of the cryptic polyA site. *Htt* sequences are shown with the core sequence of the cryptic polyA site underlined. The polyA tail is shown in bold, sequences introduced by the UAPdT primer are shown in italics and vector sequences in lower case. (**C**) 3'RACE analysis of the site at 1145 bp (◄) showed that it was not used in the minigene lines.

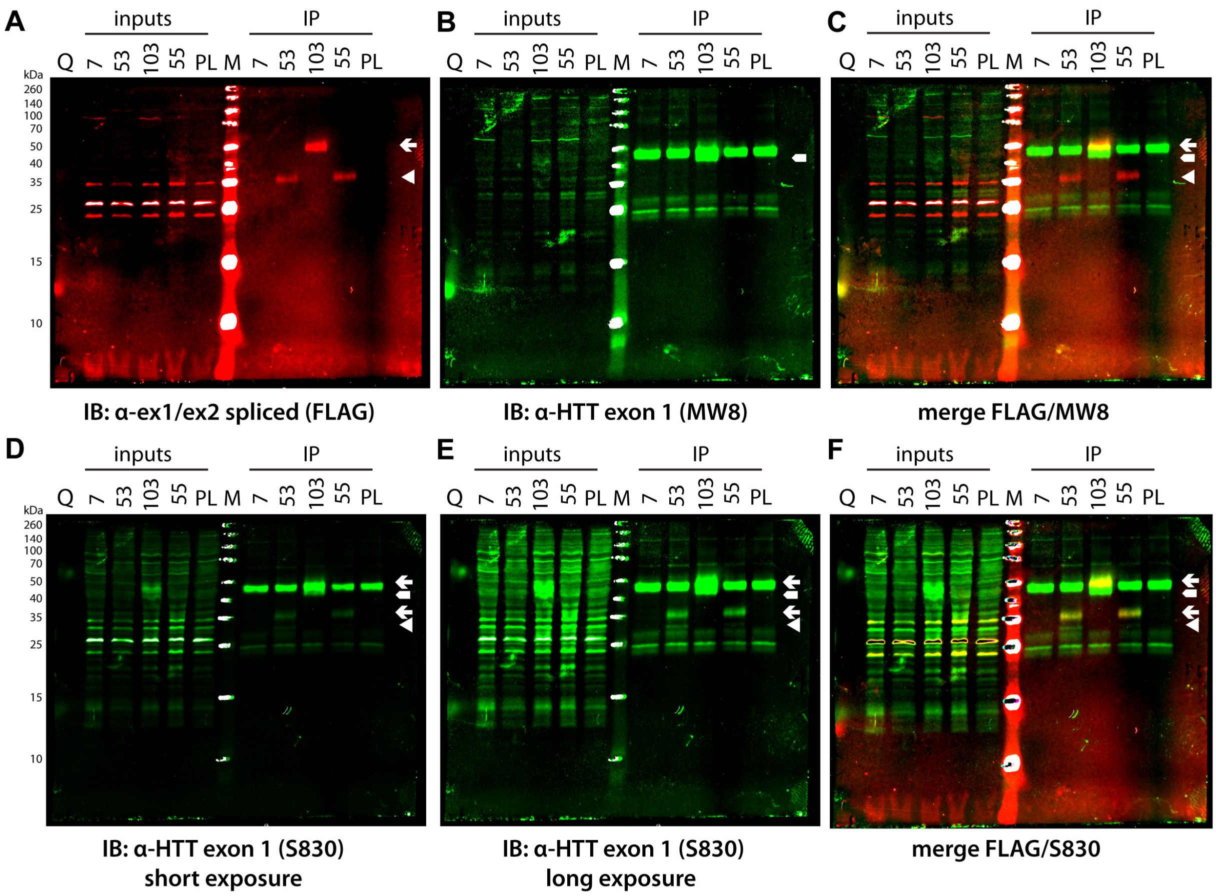

Supplementary Fig. 2. Correctly spliced, as well as exon 1 HTT protein fragments are expressed from the minigene constructs.

HTT fragments were immunoprecipitated (IP) with 3B5H10 coupled magnetic beads and immunoprobed (IB) with antibodies, as indicated, after western blotting (see also the corresponding supplementary methods section). (**A-C**) Analysis of HTT fragments. (**A**) FLAG-tag detects the properly spliced exon 1- exon 2 fragment. Q7 containing fragments were not detected because they are not efficiently immunoprecipitated with the anti-polyQ antibody 3B5H10. Q50 (🡸) and Q100 (◀) containing correctly spliced exon 1-exon 2 HTT fragments were detected. (**B**) MW8 is a neo-epitope antibody against the end of exon 1 HTT. Only a Q103 containing (⬬) fragment could be detected. MW8 does not recognize its amino acid sequence when it is present in the spliced exon 1 – exon 2 fragment (**C**) Overlay image of spliced exon 1 – exon 2 HTT (FLAG) and incompletely spliced exon 1 HTT (MW8) fragments. Note the size difference due to the inclusion/exclusion of exon2-FLAG sequences. (**D-F**) MW8 binding is conformation dependent. (**D** and **E**) An exon 1 HTT protein is also produced in the Q50 minigene lines. The S830 antibody recognized Q50 and Q100 containing spliced (🡸), as well as incompletely spliced fragments (◀ Q50, ⬬ Q100), which are apparent in the high exposure image. (**F**) Overlay image of spliced (FLAG) and exon 1 HTT (S830) containing fragments (same panel as in Fig. 1E). Note the size difference due to the inclusion/exclusion of exon2-FLAG sequences in the Q50 lines. IgG heavy and light chains resolve at 50 and 25 kDa. PL = parent line; M = PageRuler™ Prestained Protein Ladder (ThermoFisher).

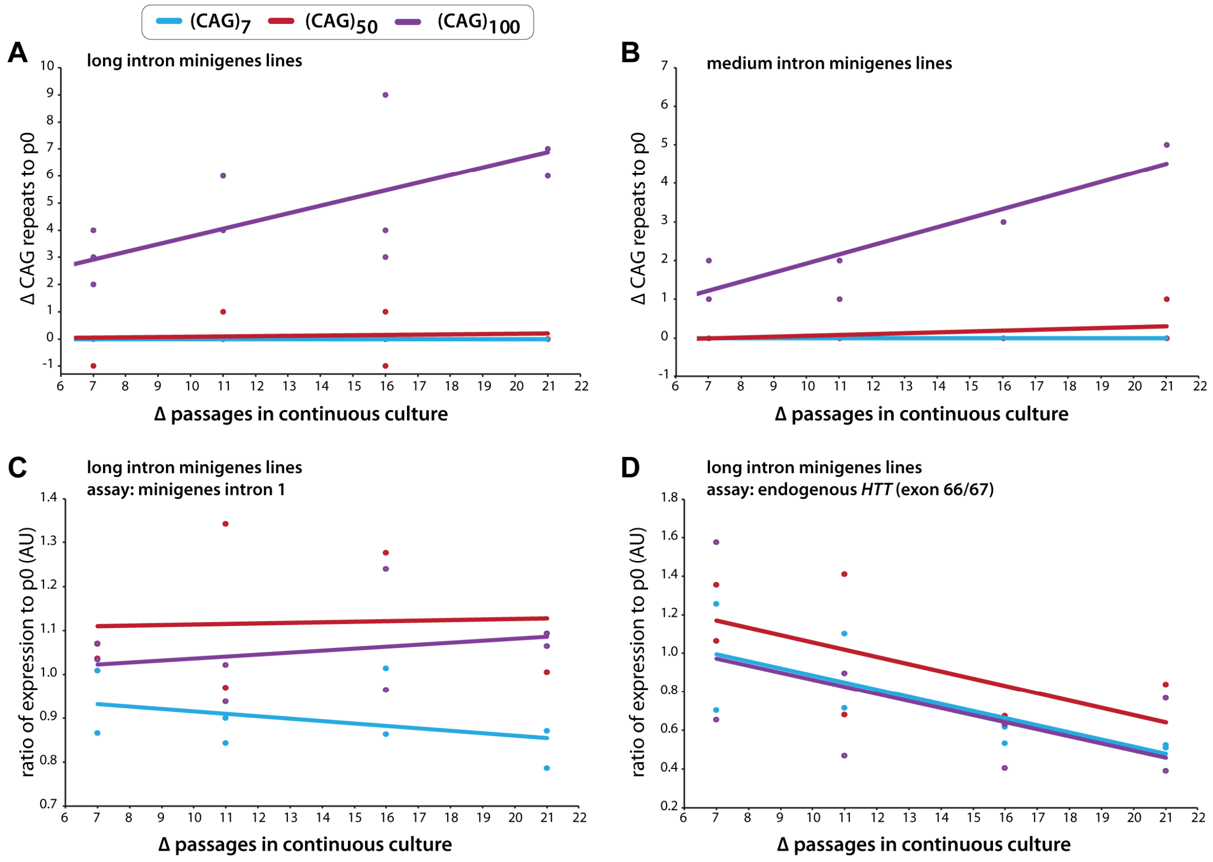

Supplementary Fig. 3. The CAG repeat expands independently of incomplete splicing and incomplete splicing is not influenced by passage number.

(**A** and **B**) Repeat instability in the long (**A**) and medium (**B**) intron minigene lines. Repeat sizes were measured at the indicated passage numbers and are shown as the difference to passage 0 (see also the corresponding methods section). Individual data points with linear regression fits are shown. N ≥ 2/CAG repeat length. Lines with $(CAG)_7$ and $(CAG)_{50}$ had stable repeats. The repeat length expanded with very similar rates over time in lines with a starting repeat length of 103 $(CAG)_{100}$ independent of the occurrence of incomplete splicing (incomplete splicing is only evident in the long intron lines). (**C** and **D**) analysis of transcript levels of incompletely spliced *HTTexon1* (**C**) and endogenous *HTT* (**D**). Individual data points with linear regression fits are shown as a ratio to the starting transcript levels at passage 0. N ≥ 2/CAG repeat length. (**C**) Incomplete splicing did not change when cells were kept in continuous passage. (**D**) The levels of endogenous *HTT* declined with continuous passage.

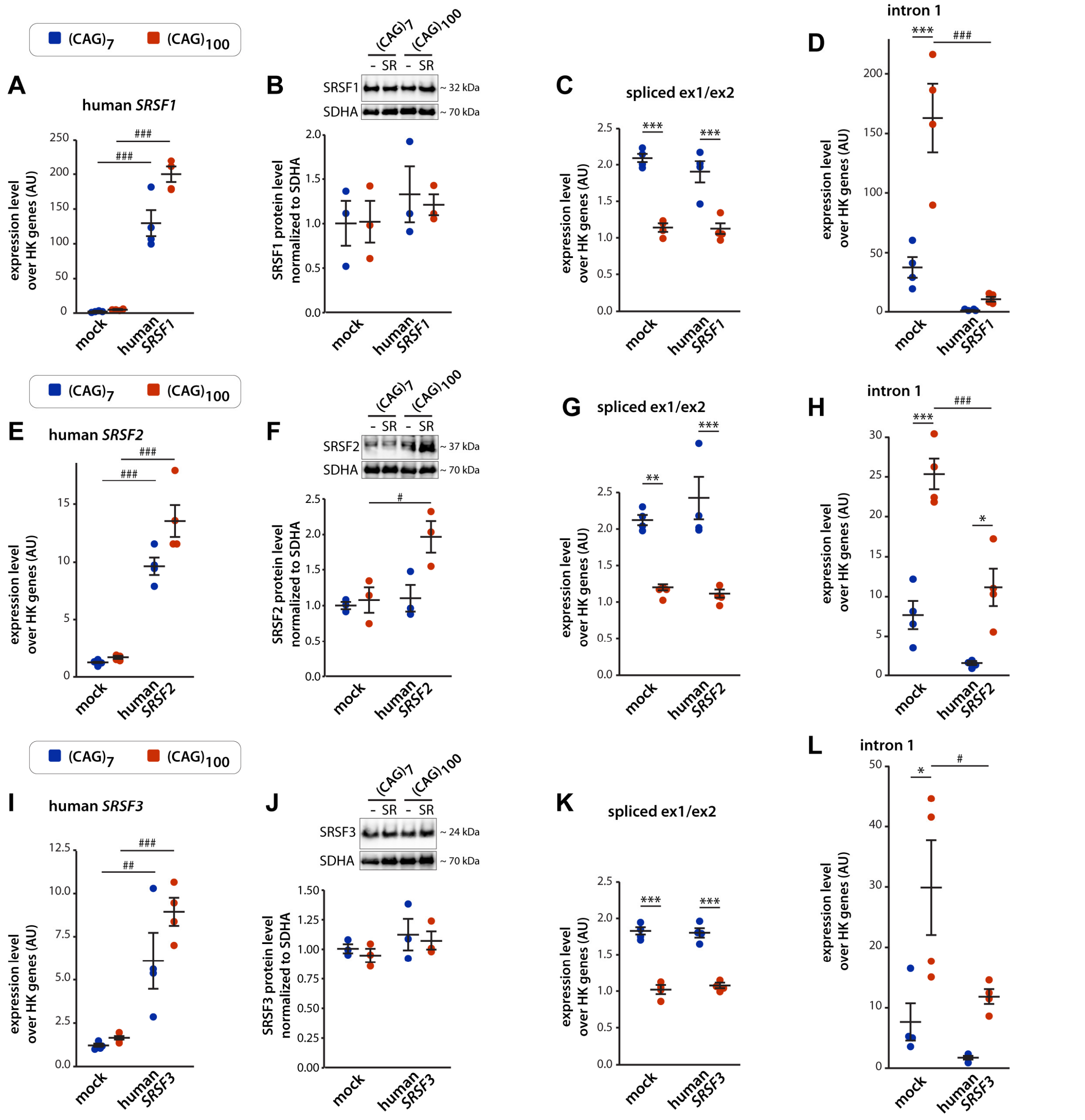

Supplementary Fig. 4. Overexpression of human splicing factors SRSF1, SRSF2 or SRSF3 does not increase the amount of incompletely spliced *Htt* minigenes

Overexpression of SRSF1 (A-D), SRSF2 (E-H) and SRSF3 (I-L) in the CAG_7 and CAG_{100} *Htt* minigene lines. All three SR proteins were significantly overexpressed in both lines at the RNA level (A, E, I) and protein level for SRSF2 (B, F, J). Uncropped blots can be found in Supplementary Fig. 8C-E. There was no effect on the canonically spliced exon 1-exon 2 transcripts (C, G, K). Overexpression of the SR proteins led in all cases to reduced levels of incompletely spliced *Htt* minigenes for both CAG repeat lengths (D, H, L). - = mock; SR = SR protein overexpression. Individual data points and the mean ± s.e.m. are shown. N = 4 independent experiments/CAG-length; two-way ANOVA with Tukey *post hoc*. SR protein overexpression effect for a given CAG-length: $^{\#}p<0.05$, $^{\#\#}p<0.01$, $^{\#\#\#}p<0.001$. Effects due to CAG-length for a given treatment: $^{*}p<0.05$, $^{**}p<0.01$, $^{***}p<0.001$.

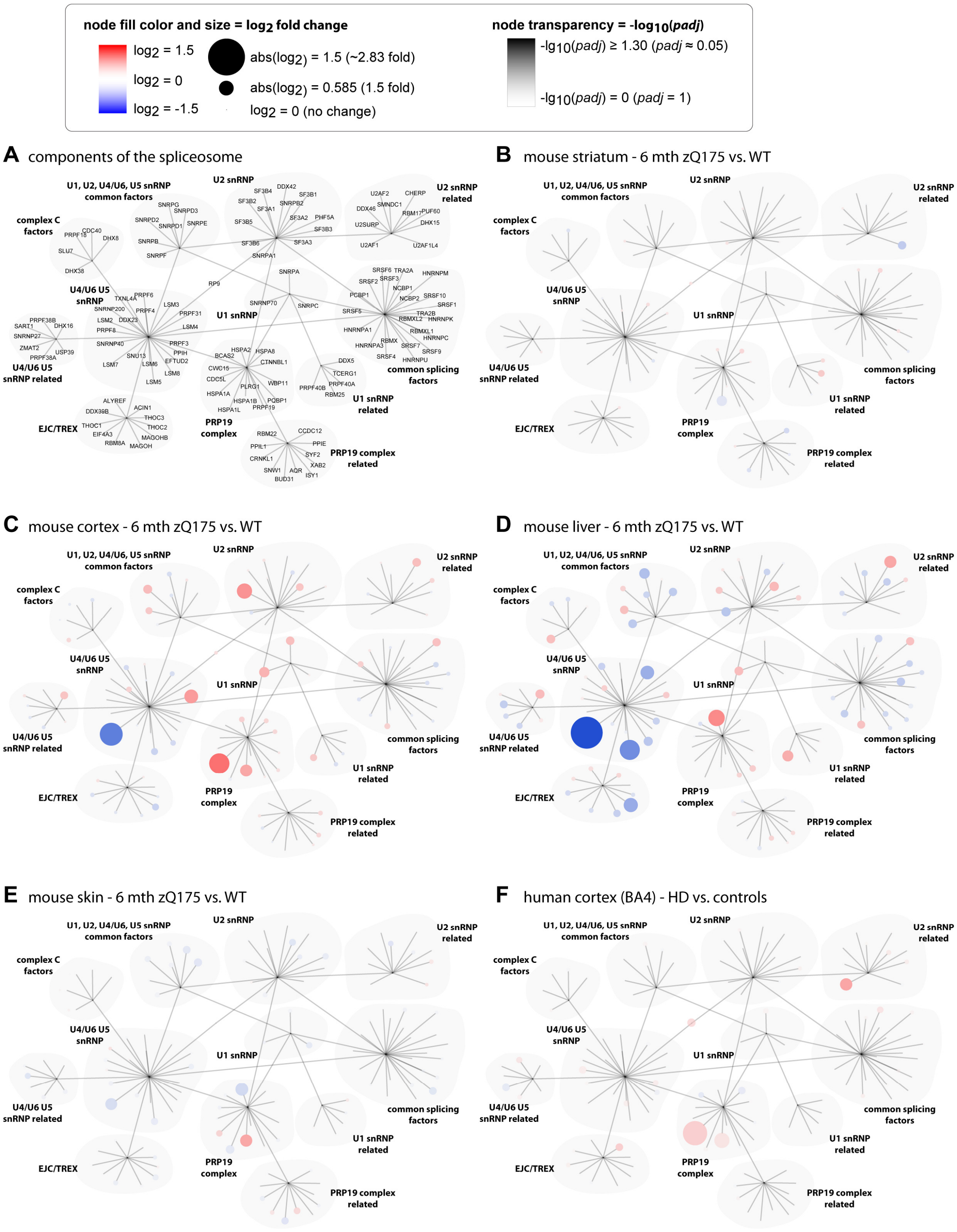

Supplementary Fig. 5. Analysis of regulation of the splicing machinery in the zQ175 mouse model and human *post-mortem* brain tissue

(**A**) KEGG pathway 'spliceosome' (mmu03040 and hsa03040) sub-complexes and their common components. Edges between sub-complexes indicate functional interaction during the splicing reaction. (**B-F**) Transcriptional dysregulation of the spliceosome as shown in (**A**) in the striatum (**B**), cortex (**C**), liver (**D**) and skin (**E**) of 6 months old zQ175 heterozygous mice and in the BA4 cortex region of HD patients (**F**). The size of the circles corresponds to the $\log_2$-fold change with the color indicating

the direction of the change. Changes with an adjusted *p*-value of less than 0.05 are indicated by reduced transparency. Only few significantly dysregulated spliceosomal components were detected and changes were not consistent between tissues. WT = wild type mice; zQ175 = heterozygous zQ175 mice; snRNP = small nuclear ribonucleoprotein complex; EJC = exon junction complex; TREX = transcription/export complex.

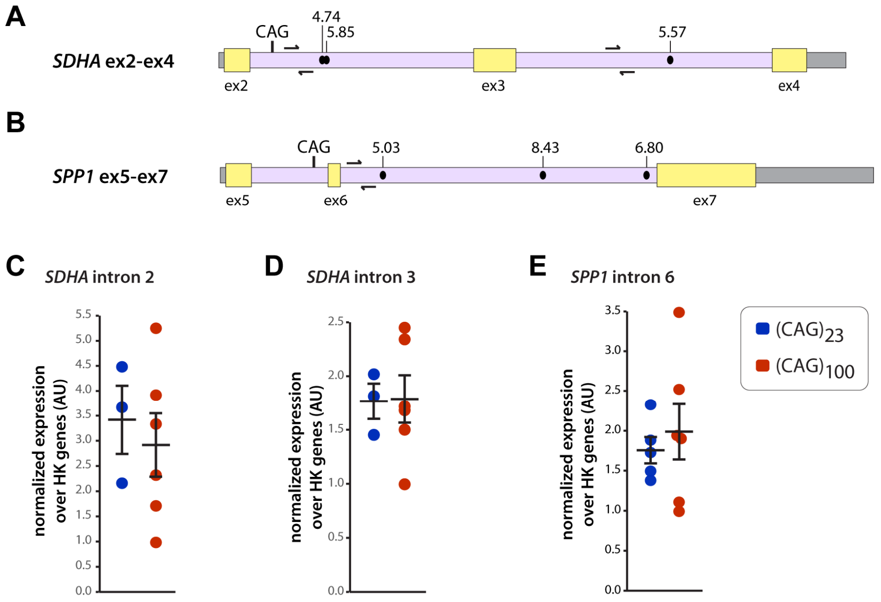

Supplementary Fig. 6. A CAG repeat is not sufficient to induce mis-splicing in other genetic contexts.

(**A** and **B**) Schematics showing the makeup of the *SDHA* and *SPP1* minigenes, respectively. The strength and location of cryptic polyA sites (●) are shown. The sites of the CAG repeat (CAG) integration are shown. (**C-E**) Analysis of retention of intronic sequences, which could indicate splicing problems. Individual data points and the mean ± s.e.m. are shown. N ≥ 3 independent cell lines/integration site (*SDHA*); n ≥ 5 independent cell lines/integration site (*SPP1*); Student's *t*-test. There were no statistically significant differences in the amount of intronic sequences when either a short $(CAG)_{23}$ or long $(CAG)_{100}$ repeat was integrated. (**C**) *SDHA* minigene intron 2. (**D**) *SDHA* minigene intron 3. (**E**) *SPP1* minigene intron 6.

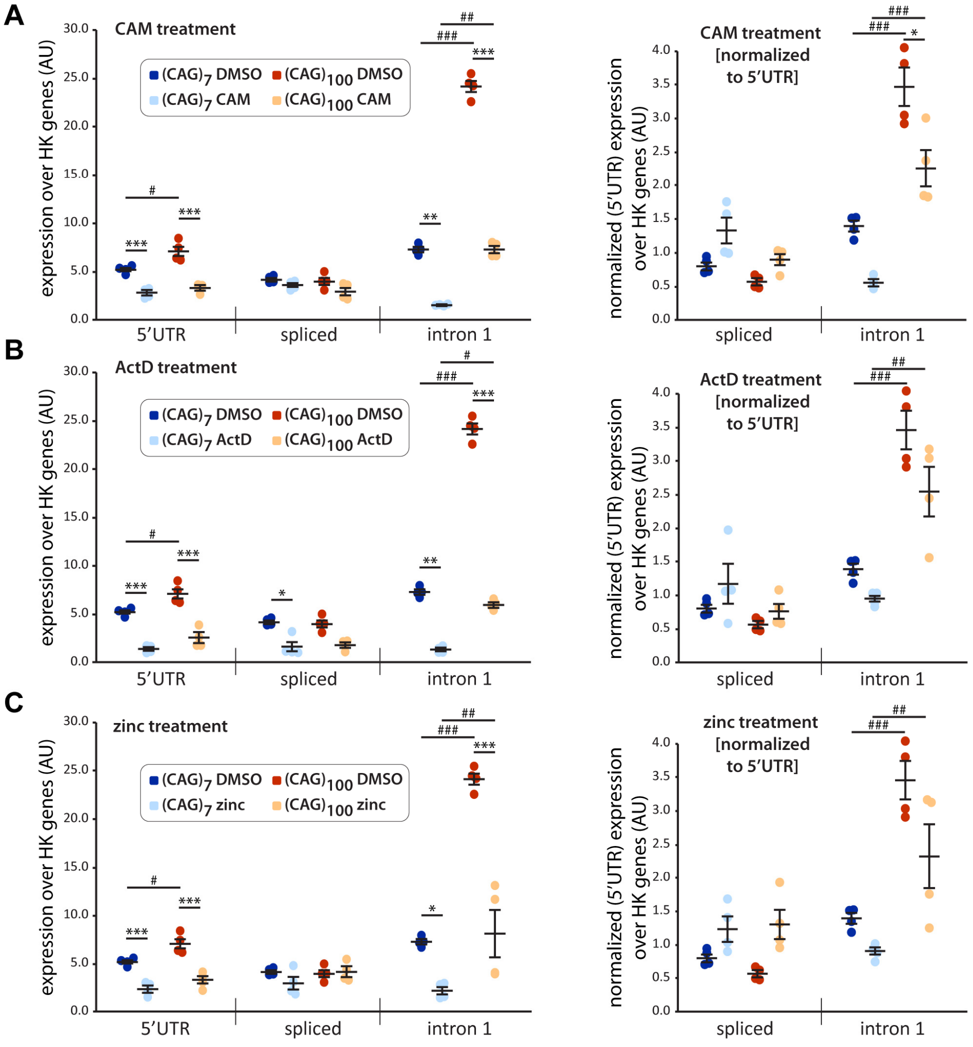

Supplementary Fig. 7. The effects of different drugs on the amount of incomplete splicing.

(**A** - **C**) Transcript analysis of drug treated samples. Left panels show the transcript levels of minigene sequences standardized to housekeeping genes (HK). Right panels show the transcript levels standardized to HK genes and normalized to the amount of 5' UTR containing sequences in the respective lines. Individual data points and the mean ± s.e.m. are shown. N = 4 independent treatments/CAG-length, two-way ANOVA with Tukey *post hoc* test: treatment: $^{*}p < 0.05$, $^{**}p < 0.01$, $^{***}p < 0.001$. Treatment x CAG-length $^{\#}p < 0.05$, $^{\#\#}p < 0.01$, $^{\#\#\#}p < 0.001$. (**A**) (S)-(+)-camptothecin (CAM) treatment. Cells were treated with 4 µM CAM for 6 hours. (**B**) Actinomycin D (ActD) treatment. Cells were treated with 0.5 µM ActD for 6 hours. (**C**) 1-hydroxypyridine-2-thione zinc salt (zinc) treatment. Cells were treated with 10 µM zinc for 6 hours.

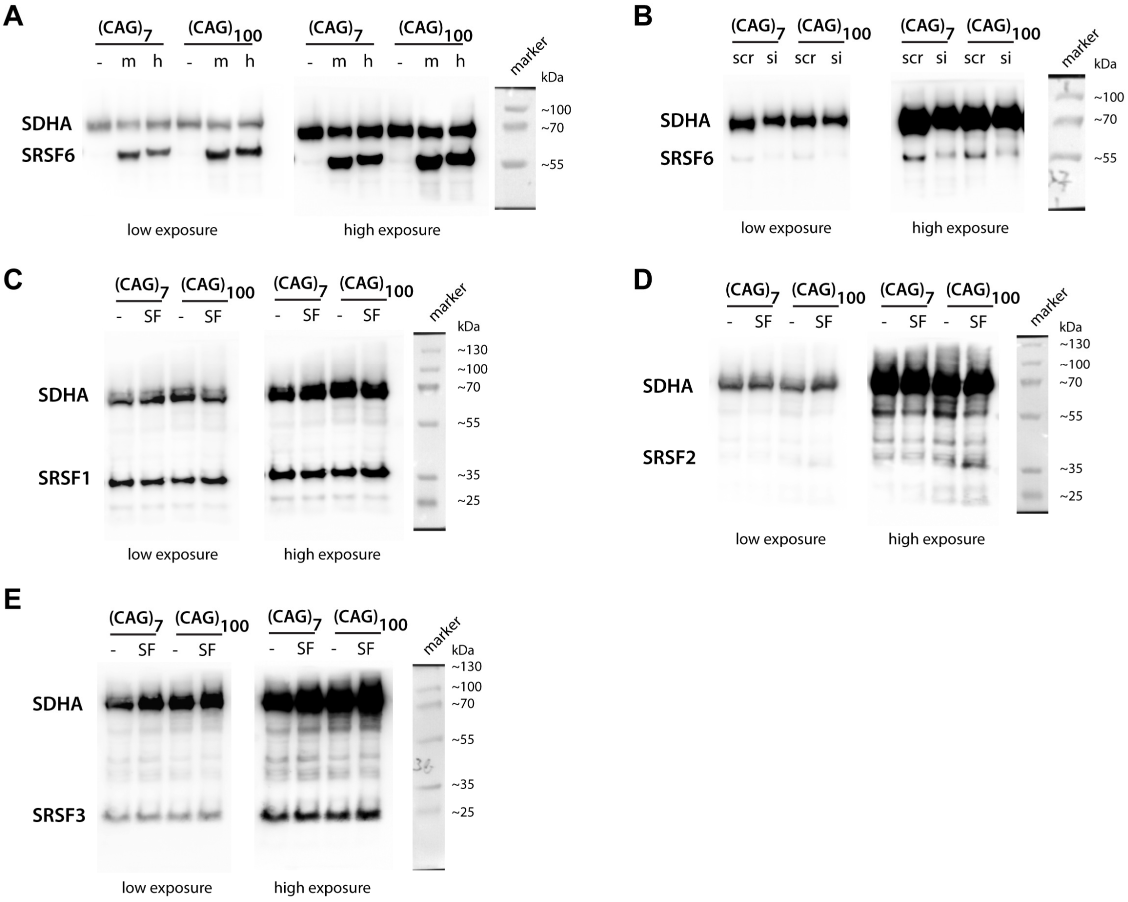

Supplementary Fig. 8. Uncropped western blot membranes.

(**A** - **E**) Uncropped images of western blots corresponding to the cropped images shown in Fig. 3 and Supplementary Fig. 4. Images are labeled in the same way. For quantification, the appropriate low or high exposure images were used. Marker was PageRuler™ Plus Prestained Protein Ladder, 10 to 250 kDa (ThermoFisher). (**A**) SRSF6 overexpression analysis as shown in Fig. 3C. (**B**) SRSF6 knock-down as shown in Fig. 3G. (**C**) SRSF1 overexpression analysis as shown in Supplementary Fig. 4B. (**D**) SRSF2 overexpression analysis as shown in Supplementary Fig. 4F. (**E**) SRSF3 overexpression analysis as shown in Supplementary Fig. 4J.

SUPPLEMENTARY TABLES

Supplementary Table 1. Oligonucleotides

name	sequence	purpose
UAPdT	GGCCACGCGTCGACTAGTACTTTTTTTTTTTTTTTTT	reverse transcription
677	GTGTGAACGCATCCAATGGG	3'RACE
677nest	GGCGTTTCATTTAGTTCGTGGT	3'RACE
1145	GCTGCTTGTGACTGGAGAGA	3'RACE
1145nest	GACCATGAGTCACAGTGCTCT	3'RACE
pHtt1_f	GCAGCGGCCGCGATTGAACTCAGAGCTTCGTGCATG	cloning of *HTT* minigenes
pHtt1_r	CGTGTTTAAACTTGGATCCTACTGGCACTACGCGG	cloning of *HTT* minigenes
pHtt2_f	GCAGGATCCAAGTCTTCAGGGTCTGTCC	cloning of *HTT* minigenes
pHtt2_r	CGTGTTTAAACTTGGTACCGCGACCCTCTGGACA	cloning of *HTT* minigenes
pHtt3_f	GCAGGTACCTCCCTGAGGCCAGG	cloning of *HTT* minigenes
pHtt3_r	CGTACGCGTGCTAGCTTCCAAAGATCTATAAGATGTCAG	cloning of *HTT* minigenes
pHtt6_f	GCAACGCGTTGCATTCCCAATACATTTATGGGAGG	cloning of *HTT* minigenes
pHtt6_r	CGTCAATTGCTACTTATCGTCGTCATCCTTGTAATCCCTGAGAGACTGTGCCACAATGTTT	cloning of *HTT* minigenes
pHtt7_f2	GCACAATTGTGTCACACTCTAGAGCCATCGAA	cloning of *HTT* minigenes
pHtt7_r2	CGTGTTTAAACCACCCCTCTAGAACCCTGG	cloning of *HTT* minigenes
pHtt30_r	GCTCCTGCAGGATATGGCTGTGACACTGGTTC	cloning of *HTT* minigenes
pHtt32_f	GCACCTGCAGGTGCATTCCCAATACATTTATGGGAG	cloning of *HTT* minigenes
CTG7	CTGCTGCTGCTGCTGCTGCTG	cloning of *HTT* minigenes
CAG7	CAGCAGCAGCAGCAGCAGCAG	cloning of *HTT* minigenes
SDHAf	GGTACCATGTGGCCAACAGTGTTGCAAACAGG	cloning of *SDHA* minigene
SDHAr	GGATCCTTTGCTCATCACCATTCTTTTGGCTG	cloning of *SDHA* minigene
SPP1f	GGTACCATGCTTTACAACAAATACCCAGATGCTGTGG	cloning of *SPP1* minigene
SPP1r	GGATCCCGGCAAGGCTATGTCAAATCCAGAT	cloning of *SPP1* minigene
BamHIF	GGATCCCGAGTCGCTCAAGTCGTTTCAGC	CAG repeat position
BamHIR	GGATCCCGGCGCCTGCGGCGGT	CAG repeat position
KpnIF	GGTACCCGAGTCGCTCAAGTCGTTTCAGC	CAG repeat position
KpnIR	GGTACCCGGCGCCTGCGGCGGT	CAG repeat position
HincIIF	GTTAACCGAGTCGCTCAAGTCGTTTCAGC	CAG repeat position
HincIIR	GTTAACCGGCGCCTGCGGCGGT	CAG repeat position
NdeIF	CATATGCGAGTCGCTCAAGTCGTTTCAGC	CAG repeat position
NdeIR	CATATGCGGCGCCTGCGGCGGT	CAG repeat position
hmSF6f-K	GCAGGATCCGCCACCATGCCGCGCGTCTACATAGGA	*SRSF6/Srsf6* overexpression
mSF6r	CGTGCGGCCGCTTAATCTCTGGAACTTGATCTGGACCTT	*Srsf6* overexpression
hSF6r	CGTGCGGCCGCTTAATCTCTGGAACTCGACCTGGAC	*SRSF6* overexpression
gRNAIa	ACACCTGGCGCCGCGTAGTGCCAGTG	gRNA I
gRNAIb	AAAACACTGGCACTACGCGGCGCCAG	gRNA I
gRNAIIa	ACACCGCTGACATCTTATAGATCTTG	gRNA II
gRNAIIb	AAAACAAGATCTATAAGATGTCAGCG	gRNA II
gRNAIIIa	ACACCGGGGGTTGTTAACTCTTGACG	gRNA III
gRNAIIIb	AAAACGTCAAGAGTTAACAACCCCCG	gRNA III
gRNAIVa	ACACCTGACCGGTCACGTAAACTGCG	gRNA IV
gRNAIVb	AAAACGCAGTTTACGTGACCGGTCAG	gRNA IV

Oligonucleotides were purchased from Eurofins MWG.

Supplementary Table 2. qPCR assays

assay	forward primer	reverse primer	probe (5'-FP, 3'-Q)	supplier	comment
endogenous *HTT*	TGCTTCTTTGTCAGCG CGT	CTCCAGCTTGCCCATCCT	CTGATGACATGTGGGAGGATCGCC (Cy5.5/BHQ3)	Eurofins MWG	
minigene 5' UTR	CTGAGCGCCTTGGTTC C	ATCCTACTGGCACTACGC	CGCCACTTAGCAGCAAGGCAATGAAT (Cy5.5/BHQ3)	Eurofins MWG	58°C
minigene spliced ex1/ex2 -19f	AGGAACCGCTGCACC GA	ACTTATCGTCGTCATCCT TGTAATC	AGAAAGACCGTGTGAATCATTGTCTAAC AATATGTGA (FAM/TAMRA, TexasRed/BHQ2, Cy5.5/BHQ3)	Eurofins MWG	
minigene spliced ex1/ex2 -34f	TGCCAGGTCCGGCAG AG	ACTTATCGTCGTCATCCT TGTAATC	AGAAAGACCGTGTGAATCATTGTCTAAC AATATGTGA (FAM/TAMRA, TexasRed/BHQ2, Cy5.5/BHQ3)	Eurofins MWG	
minigene intron 1	CTTGCGGGGTCTCTGG C	TCAGCGAGTCCCTGGCT G	CCTCAGAGGAGACAGAGCCGGGTCA	Eurofins MWG	published in [1] as 'early intron 1'
mouse Srsf6	AAGCCAAGAACAAGC CATAGGC	CTCCTACTCCGAGACCTT CTT	TCCTACTCTGGCAGTAGATCCAGATCA (FAM/TAMRA)	Eurofins MWG	
human SRSF6	CAAAGGCCCGTTCTGT GTCC	GGAACTCGACCTGGACC TTG	CTTGACTTTGAGCGAGATCTAGAACGG (Cy5.5/BHQ3)	Eurofins MWG	
human SDHA intron 2	GAGTCATAGACATAAG GGAAAAATTTCT	CTATGTTTATATTTGTACT TGCAATTAAGTATT	TAGGTATCCAATGCATGCTGAAATTATTT TCAGTGTA(FAM/TAMRA)	Eurofins MWG	
human SDHA intron 3	GGAGATCACCTAAAG AGGAGGAA	AAGCACAGGGACAAATG AAACCC	CAGAATTTAGGTTGGGGAAGAAAAGGG C (FAM/TAMRA)	Eurofins MWG	
human SPP1 intron 6	GTAAGTTCTCATTTTCA ATCAGAGGC	ATTTCATCAGAAGAGAAT CCAGGCA	CATCATGCCTTGAAGAGATGAAAGAAGG C (FAM/TAMRA)	Eurofins MWG	
ChIP 1011	GACGCTCAAAACATTT CCTGGA	TGCCCAGAGTTGAGAGA AAGGA	CCATGCTGAGTGTTGAGCCCTGTGC (FAM/TAMRA)	Eurofins MWG	
ChIP 1408	CTTGACCTGAACTCAG AGAGAT	ACTTGGACAGCCCAGCA TG	ATCCTCCTGCCTCTGCCTCCTC (TxRED/BHQ2)	Eurofins MWG	
ChIP 2019	AAACAGCTGAGTAAA ATGGCAAGTG	CAGTGTTCTTAGATTCCA GATAGCA	TTAATTGTTTACTGAATGGGTTAAGCTCC TTG (Cy5.5/BHQ3)	Eurofins MWG	
ChIP 2722	AATGAAAACATCTCCC TCCCCATTT	GCCCAAATTTTATCTGGA AGGACAA	CTAACTTTGTACCCAGGAGCAAGCTCT (TxRED/BHQ2)	Eurofins MWG	
ChIP 3015	TAACAAACTCACTGCT TGGAAGCTT	CTGTTTTGGCTGTTCTCA TTTCGAA	ATTTTTGGCATTTTGTCCACTTGACTCAGT TAAAAG (Cy5.5/BHQ3)	Eurofins MWG	
ATP5B				Primer Design[1]	
SDHA				Primer Design[1]	
ACTB				Primer Design[1]	

All assays were run at 60°C extension temperature unless otherwise specified. [1]No information is disclosed for housekeeping genes by PrimerDesign. FP = fluorophore; Q = quencher

Supplementary Table 3. Regression fit statistics

Figure	Dataset		Regression type	R^2	ANOVA *F*	ANOVA Significance *F*
Fig. 2C	endogenous HTT (exon 66/67)	short intron	linear	0.0489	0.6170	0.447405
		medium intron	linear	0.0065	0.1376	0.714381
		long intron	linear	0.0339	1.7191	0.195927
		exon 1 only	linear	0.4013	3.3510	0.126673
Fig. 2D, 2F, 2H, 2J	spliced exon 1-2	short intron	power	0.0451	0.5666	0.466138
		medium intron	power	0.0356	0.8481	0.366640
		long intron	power	0.0725	3.8313	0.056012
Fig. 2E, 2G, 2I, 2K	intron 1	short intron	polynomial (3rd order)	0.6793	7.0617	0.007853
		medium intron	polynomial (3rd order)	0.0335	0.2313	0.873488
		long intron	polynomial (4th order)	0.4794	10.8180	0.000003
		exon 1 only	polynomial (4th order)	0.4791	0.9402	0.573822
Fig. 5A	ChIP – $(CAG)_7$		polynomial (4th order)	0.9970	245.9812	0.000418
	ChIP – $(CAG)_{100}$		polynomial (4th order)	0.9274	9.5743	0.046815
	IgG – $(CAG)_7$		polynomial (4th order)	0.1680	0.1515	0.950135
	IgG – $(CAG)_{100}$		polynomial (4th order)	0.5831	1.0491	0.504601
Fig. 5C	$(CAG)_7$		polynomial (2nd order)	0.8588	3.0410	0.375771
	$(CAG)_{100}$		polynomial (2nd order)	0.8419	2.6626	0.397614
Fig. 5D	$(CAG)_7$		polynomial (2nd order)	0.8732	3.4444	0.356035
	$(CAG)_{100}$		polynomial (2nd order)	0.9986	353.2270	0.037597
Fig. 5E	$(CAG)_7$		polynomial (2nd order)	0.9806	25.2194	0.139430
	$(CAG)_{100}$		polynomial (2nd order)	0.9990	488.6767	0.031971
Fig. S3A	$(CAG)_7$		linear	0	0	0
	$(CAG)_{50}$		linear	0.0086	0.0203	0.899723
	$(CAG)_{100}$		linear	0.5498	4.7227	0.161848
Fig. S3B	$(CAG)_7$		linear	0	0	0
	$(CAG)_{50}$		linear	0.2225	3.4467	0.204509
	$(CAG)_{100}$		linear	0.8798	18.9742	0.048872
Fig. S3C	$(CAG)_7$		linear	0.1508	1.2388	0.381539
	$(CAG)_{50}$		linear	0.0022	0.0066	0.942480
	$(CAG)_{100}$		linear	0.0777	4.1656	0.178039
Fig. S3D	$(CAG)_7$		linear	0.5379	22.7596	0.041239
	$(CAG)_{50}$		linear	0.3633	3.5324	0.200942
	$(CAG)_{100}$		linear	0.2819	4.1656	0.178039

SUPPLEMENTARY REFERENCES

1 Sathasivam, K. *et al.* Aberrant splicing of HTT generates the pathogenic exon 1 protein in Huntington disease. *Proc Natl Acad Sci U S A* **110**, 2366-2370, doi:10.1073/pnas.1221891110 (2013).
